# Positive Psychological Coaching Tools and Techniques: A Systematic Review and Classification

**DOI:** 10.3389/fpsyt.2021.667200

**Published:** 2021-07-09

**Authors:** Stefanie Richter, Llewellyn E. van Zyl, Lara C. Roll, Marius W. Stander

**Affiliations:** ^1^Faculty of Psychology, Technische Universität Dresden, Dresden, Germany; ^2^Human Performance Management, Department of Industrial Engineering, University of Eindhoven, Eindhoven, Netherlands; ^3^Optentia Research Focus Area, North-West University Vaal Triangle Campus, Vanderbijlpark, South Africa; ^4^Department of Human Resource Management, University of Twente, Enschede, Netherlands; ^5^Department of Social Psychology, Institut für Psychologie, Goethe University, Frankfurt am Main, Germany; ^6^Department of Applied Psychology, Lingnan University, Tuen Mun, Hong Kong; ^7^Department of Work, Organisational and Personnel Psychology, KU Leuven, Leuven, Belgium

**Keywords:** positive psychological coaching, positive psychological tools, positive psychological techniques, positive psychological interventions, strengths based coaching

## Abstract

Positive psychological coaching (PPC) has emerged as a popular “paradigm” for practitioners interested in the professional development of people. A recent review consolidated the literature on PPC and produced a 5-phase positive psychological coaching model aimed at facilitating professional growth. However, little is known about practically operationalizing each phase of the coaching process (i.e., how to facilitate each phase and which underlying tools and techniques could be employed to do so). As such, the purpose of this systematic review was to address this limitation by (a) determining which coaching tools and techniques are proposed within the coaching literature and (b) classifying the identified tools and techniques into the respective phases of PPC model. The investigation used a two-step approach by conducting a systematic literature review (to identify various PPC tools/techniques) followed by an iterative heuristic classification process (to assign these PPC tools/techniques to a known PPC model). The systematic literature review resulted in 24 peer-reviewed publications on positive psychological coaching, providing 117 different coaching tools that could be condensed into 18 overarching coaching techniques. The iterative classification process showed that most techniques and tools are useful in at least two phases. Interestingly, experts still vary in opinion on the timing and application of these specific techniques and tools within the positive psychological coaching process. This study provides researchers and practitioners with practical guidelines to facilitate a positive psychological coaching process.

## Introduction

Positive psychological coaching (PPC) has emerged as a popular “paradigm” for practitioners interested in the professional development of people (1). This popularization was fuelled by the scientific advancements in positive psychology in the early 2000's, and lauded as a new approach to optimize the potential of people through focusing on “what already works well,” rather than on “fixing what is wrong” (2, 3). This approach seemed to be favored by both practitioners and clients as it promotes growth, optimizes psychological strengths, and shifts the focus away from addressing psychopathology or professional inefficiencies (4, 5). From this perspective, it positions personal growth and goal achievement as a function of the identification, awareness, and active utilization of one's signature strengths (6, 7). This, in turn, largely removes the stigma attached to the use of psychological services and lowers the threshold for utilization/participation (8). It is, therefore, not surprising that PPC has become a buzzword within the modern-day coaching practitioner's lexicon (9).

Despite its rapid adoption in practice, the formalization of positive psychological coaching as a scientific concept, a sub-discipline of positive psychology or a “paradigm” is still in its infancy (10). Even though the concept's origins can be traced back to a chapter by Kauffman and Scoular almost two decades ago (6), the scientific discourse on PPC is still largely centered around its conceptualization (10), the differentiation between other types of coaching approaches (1) or how it differs from counseling, mentoring, and therapy (11, 12). A study has shown that at least 24 different definitions of PPC exist in the scientific literature, with less than a 20% overlap in common elements between these definitions (10). From these definitions, a myriad of positive psychological coaching approaches or models have been developed ranging from authentic happiness coaching (13) and strengths-based coaching (8) to appreciative inquiry coaching (14) and quality of life coaching (15). Each coaching model provides a different means to distinguish itself from others, which further distracts from what fundamentally constitutes PPC and what tools or techniques are considered “positive.”

Some authors argue in favor of this inconsistency and state that “*definitions* (and approaches) *seldom stay static, unless the area has stagnated*” [(16), p. 3]; implying inconsistency and variety signifies growth or development in the discipline. Whereas, others argue these inconsistencies in fundamental components of a scientific concept signify an invalid concept or that it leads to a fragmentation in the scientific discourse on the subject matter (17). Some level of agreement in the fundamental principles of a scientific concept is therefore required to ensure that a discipline can develop and that it can lead to critical discrimination, an exploration of its function within the larger psychological system, and empirical verification (17, 18). For PPC to distinguish itself from other approaches to coaching and to develop as a science, there needs to be an objective, generally accepted, well-researched, organized body of knowledge supporting PPC's scientific identity (10, 19). The lack of a standardized approach may not only have negative implications for the discipline but may adversely affect the effectiveness of PPC interventions as they are not built on validated evidence-based theoretical frameworks (20).

**Figure 1:**
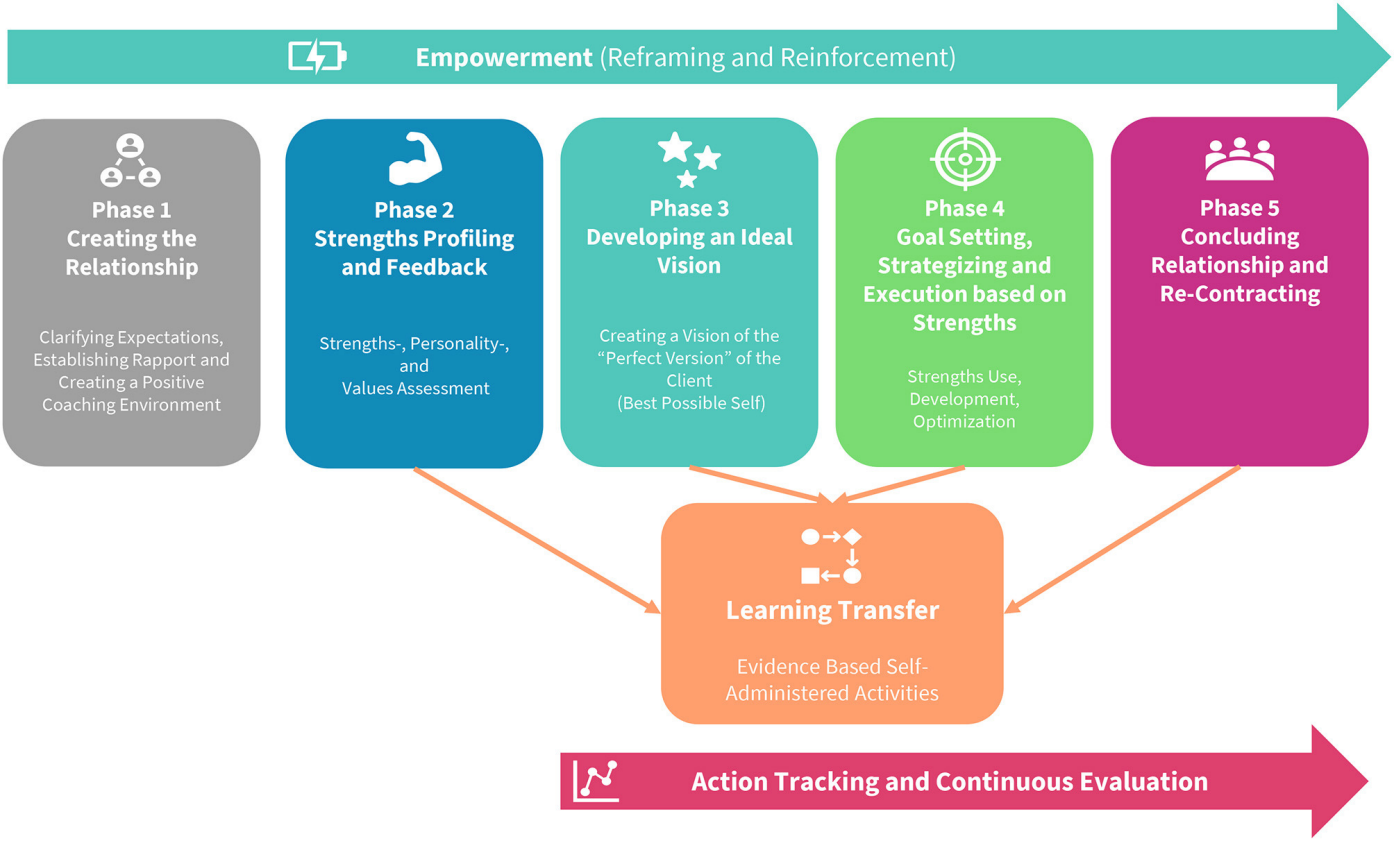
The positive psychological coaching model (PPCM) (10).

A recent systematic literature review by Van Zyl et al. (10) aimed to consolidate the literature on PPC to develop an integrated definition and model thereof. They found that PPC can be defined as “a *short- to medium-term professional, collaborative relationship between a client and coach, aimed at the identification, utilization, optimisation and development of personal/psychological strengths and resources in order to enhance positive states, traits and behaviors. Utilizing Socratic goal setting and positive psychological evidence-based approaches facilitate personal/professional growth, optimal functioning, enhanced well-being, the actualization of people's potential and aid in coping with work-demands.”* [(10), p. 11]. From this definition and the common elements of other PPC models, the authors constructed a clear, demarcated coaching model comprised of five sequential coaching phases, supported by three continuous processes (c.f. Figure 1).

Van Zyl et al. (10) found that a PPC process follows a logical and sequential chronological order: First, a professional positive relationship between the client and coach is established. Here, the coach attempts to establish rapport and engages in various activities such as job shadowing to understand the nature of the clients work and how he/she fits into the organization's vision (*Creating the Relationship*). Second, the coach employs both strengths-focused psychometric assessments and other strengths-spotting techniques to identify the client's signature strengths. Here the aim is to provide the client with feedback on his/her strengths and to facilitate the development of a mindset of strengths use (*Strengths Profiling and Feedback*). Third, the client is facilitated to explicitly develop a clear picture of his/her perfect future self, where all dreams have been achieved and where he/she is living in an optimal range of positive functioning (*Developing Ideal Vision*). Fourth, the client identifies strategic goals centered around his/her strengths and develops action strategies that would bring him/her closer to the ideal vision (*Realistic Goal Setting, Strategizing And Execution Around Strengths*). Finally, the client is prepared for terminating the relationship and a discussion on re-contracting occurs. Here all parties reflect on the effectiveness of the coaching process and whether all goals were met. These phases are supported by three continuous processes that (a) aid in transferring the skills learned during the coaching process into the work environment (*Learning Transfer*), (b) continuous evaluation of the effectiveness of the coaching process through tracking both goal achievement and well-being (*Action Tracking and Continuous Evaluation*), and (c) empowering the client to achieve goals, reinforcing strengths, and helping to frame meaning (*Empowerment*).

Although Van Zyl et al. (10) addressed the need for a consolidated definition- and a process-orientated positive psychological coaching model, it is unclear how each phase of the model can be operationalised in practice. Operationalisation of a theoretical model occurs when it is clear (a) how each phase or component of the model is defined, (b) the sequential order of such is established, (c) how/why components in a model relate, and (d) what approaches, tools or techniques are required to activate each component (21). Once all four of these factors are theoretically confirmed, a model can be subjected to empirical validation. Van Zyl et al. (10) addressed the first three of these components but failed to describe the underlying tools or techniques required to facilitate each phase of the model. From this perspective, coaching tools are broadly defined as instruments or measures that have set procedural guidelines that have been validated for use within a given coaching context (22). This may include psychometric assessment measures or validated self-administered intentional activities. Coaching techniques, on the other hand, refer to a specific technique such as a skill, ability or competence, which a coach could employ during the coaching process (23). Within the PPC literature, very little evidence exists about the specific tools and techniques that are explicitly applicable or considered “positive.” Further, no clear differentiation between the tools or techniques are made and thus used interchangeably in PPC research (22). Additionally, it is also not clear from the literature which practical tools or techniques positive psychological coaches employ to support the growth of their clients.

Although, no broad classification framework for PPC tools/techniques exists, there are mentions of specific tools and techniques that positive psychological coaches could employ to facilitate the development of clients. Specific tools that are mentioned in the literature pertain to the use of strengths-based psychometric measures to identify the strengths of clients (e.g., the VIA signature strengths inventory; the Realise2; the Skills Finder 2.0), as well as various self-administered intentional activities (or “self-help tools”) used to support the development of positive states/behaviors (e.g., the gratitude visit). Other techniques primarily relate to the use of behavioral strategies or psychological cues that facilitates the establishment of rapport [e.g., SOLER; (24)] or reframing techniques that aid clients to translate their narratives from victims to survivors (25). Despite such, no academic literatures exists that provide a comprehensive guide to, nor explanation of, the tools/techniques positive psychological coaches do or could employ during the coaching process (10, 26). This problem is further fuelled by the lack of clear coaching intervention protocols published alongside empirical coaching manuscripts (27). Therefore, it is unclear which tools/techniques are readily available for positive psychological coaches to use within the coaching process, nor in which phase of the coaching process their use would be the most appropriate.

As such, the purpose of this paper was to identify which positive psychological coaching tools and techniques positive psychological coaching researchers employ and how such can be classified into the various phases of Van Zyl et al.'s PPCM (10). First, a systematic review was used to determine which coaching tools and techniques are suggested in the literature. Second, an iterative heuristic classification process was employed to classify each of the identified tools/techniques into the respective phases of the PPCM. This study aims to further the operationalisation of PPC as both a practice domain and scientific framework.

## Methods

### Research Approach

The research approach consisted of a systematic literature review (to identify PPC tools/techniques), followed by a three-step, iterative heuristic classification process to classify the most commonly used coaching tools/techniques into the various phases of Van Zyl et al.'s PPCM (10).

A systematic literature review was deemed appropriate as it aims to synthesize an answer to a clearly defined research question. This is done through systematically identifying, selecting, and critically evaluating available research on a certain topic (28). In the present systematic review, the “Preferred Reporting Items for Systematic Reviews and Meta-Analyses (PRISMA)” guidelines were followed (29). The PRISMA guidelines aim to enhance transparency, clarity, and credibility by providing a universally accepted evidence-based checklist of components, which are reported within the systematic literature review. The PRISMA checklist is provided in S2 Checklist. Following those guidelines, a clear extraction and classification taxonomy aligned to the purpose of this study was developed and systematically applied (28).

Once the tools and techniques were identified, an iterative heuristic classification process was followed to classify findings into the different phases of the coaching mode. The first step involved an independent classification by all four authors, the second consisted of independent classifications by six experts in the field of coaching psychology and in the third step all ratings were combined and discussed, resulting in the final classification based on specified criteria.

### Search Strategy

**Figure 2:**
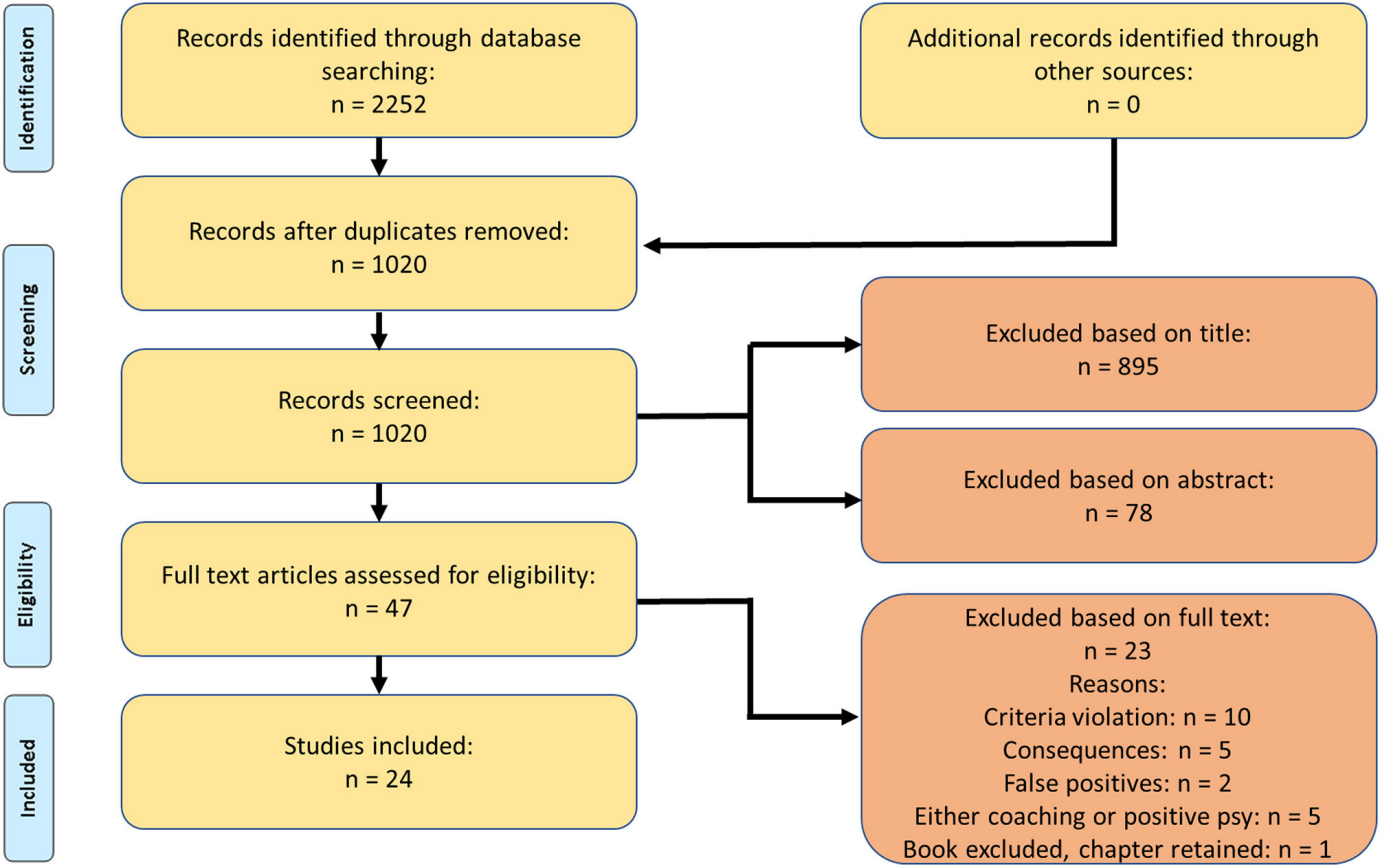
Flow chart of studies identified, included and excluded.

Between April and June 2019, a comprehensive systematic literature search was conducted in the following bibliographic databases: ACM Digital Library, PsychInfo, Scopus, ScienceDirect, and Web of Science. A total of nine primary search terms were entered: “positive psychology coaching,” “strengths coaching,” “strengths-based coaching,” “positive coaching,” “positive therapy at work,” “positive personal development,” “integrative positive coaching,” “well-being coaching,” and “happiness coaching.” After first applying the primary search term, we subsequently and secondly conducted a search with a combination of each primary term with the secondary terms “model OR process OR theory OR program” (e.g., “positive psychology coaching” AND “model OR process OR theory OR program”). We identified 2252 titles from the year 2000 up until June 2019 (Figure 2 shows the flow diagram of the article selection). The year 2000 marked the start of the positive psychology paradigm. Therefore, this starting point for the literature search seemed most appropriate.

### Eligibility Criteria

This review aimed to identify *academic* peer-reviewed, theoretical articles with a focus on positive coaching psychology. Specifically, for *inclusion* in the present paper, manuscripts needed to be (1) academic peer-reviewed, theoretical articles, books, or book chapters focusing on model- or theory construction, (2) centered around positive coaching psychology, (3) aligned with the theoretical approach of positive psychology but could emerge from any field of application (e.g., sports or business), (4) written in English, (5) published in journals that were ISI, Web of Science and Scopus listed, (6) published between 2000 and June 2019, and (7) needed to mention specific coaching tools/techniques.

*Excluded* were all manuscripts that were (1) published in languages other than English; (2) non-peer-reviewed books and articles (e.g., popular psychology or management books), (3) manuscripts focusing on developing instruments, empirical work or coaching intervention validations, (4) unpublished master and doctoral theses, (5) textbooks and conference proceedings, (6) publications with a focus on non-psychological and/or non-behavioral coaching (e.g., physical strengths conditioning in professional sports), (7) publications with a focus on positive coaching outcomes, and (8) publications that do not include the specific use of tools/techniques/interventions^1^.

### Study Selection

Following the systematic literature search, studies were selected in four distinct phases involving all four authors. In the first phase, we screened all titles against the inclusion and exclusion criteria. In the second phase, the abstracts of those publications included in the first phase were extracted and screened. The full papers were screened in the third phase, and a final decision was made regarding their inclusion. In the fourth and final phase, the complete list of identified publications was sent to five academics, who are experts in positive psychology and positive coaching psychology, for review. They did not suggest additional publications that met our eligibility criteria.

The initial systematic literature search provided 2,252 hits (Figure 2). Once duplicates were removed, 1,020 unique titles were revealed. A total of 895 publications were removed based on titles, 80 publications after screening their abstracts, and 21 manuscripts after reading the full texts. Ten manuscripts were excluded based on violating the inclusion criteria (e.g., empirical intervention studies). Further, five articles were excluded because the focus was specifically on the consequences of positive psychology coaching. Four papers were excluded as the focus was either on general coaching or on positive psychology, but not on their combination of the two. Two abstracts were initially selected because they contained our keywords. Yet, they were later excluded when the main text did not explicitly discuss such and was subsequently classified as false positives. In one book, only one chapter was relevant and hence the remaining chapters were excluded. As such, 24 articles were identified and included in the final selection. Each of these 24 articles was reviewed and the tools, techniques or interventions mentioned were extracted. All 24 articles were deemed relevant for further analysis (see Appendix).

### Data Recording and Analyses

Verbatim data from the included studies were extracted and captured onto a Microsoft Excel Spreadsheet. Subsequently, thematic content analysis (30) was applied because this procedure allowed the quantification of large amounts of textual information (31). This procedure has the advantages that (a) it is non-intrusive (32), (b) flexible (30), and (c) allows for the replication and quantification of results in terms of frequencies and percentages (Van Zyl, 2013). Still, this analysis carries the same types of limitations as traditional nominal-oriented measurement techniques (33).

For the data analysis, we followed best practice guidelines as outlined by Miles and Huberman (34), who recommend the following six steps: First, all researchers read all included articles to get an overview and make initial notes (30). Second, when working through the data set, initial codes are generated based on data features. Third, those codes are clustered into potential themes according to their similar characteristics. Fourth, the researchers revisited the themes to generate a thematic map based on the frequency of occurrence. Fifth, analyses are refined to ensure coherence between definitions, names and labels for each theme. Sixth, the themes are combined based on their frequency of occurrence. Following these steps, the researchers derived the most common techniques or tools employed in positive coaching presented in Table 1.

**Table 1:** Common positive psychological coaching tools and techniques.

**Technique (frequency)**	**Brief description**	**Positive psychological coaching tools**
Providing self-administered intentional activity *f* = 15	Providing clients with brief evidence-based positive psychological intervention strategies to aid in developing positive states, traits and behaviors through encouraging deliberate practice outside the coaching sessions (e.g., Gratitude visit).	• Gratitude visit • Writing letters of gratitude • Counting one's blessing • Practicing optimism • Performing acts of kindness • Using one's strengths in new ways • Affirming one's most important values • Meditating on positive feelings toward self and others • Visualizing ideal future selves • Recommending books from positive psychology researchers • Positive journaling • Generating a favor bank • Three good things activity • Daily savoring task • Best-possible self-activity • Using an optimism-building exercise • Having a strengths date: choosing a companion and identifying as well as utilizing strengths together • Practicing active, constructive responding • Planning a day with a personally enjoyable activity or an altruistic activity • Creating a strengths family tree • Writing a life summary • Reflecting on the meaning of daily activities • Keeping a gratitude journal • String of pearls intervention • Strengths-based journaling
Strength-focused psychometric assessments *f* = 15	Employing various forms of psychometric assessments, or simulations exercises, to identify the manifested strengths of the client (e.g., VIA Signature Strengths Inventory).	• Positive 360-degree evaluation • VIA Signature Strengths Inventory • Projective techniques • Gallup-Clifton Strengths Finder 2.0 • Realise2 • Assessing and tracking positive states, traits and strengths.
Guided self-reflection *f* = 14	Employing validated tools to aid the client in systematically discovering his/her own potential, strengths or solutions to manifested problems. Insights are developed under the supervision of a coach (e.g., History of the Future Exercise).	• Sense checking—status checking • Reflect upon previous achievements and accomplishments • Appreciative questions to encourage individuals to relive past events where they felt hopeful • “Developing a history of the future” to help clients describe their picture of success by identifying their legacy • Envision himself in the shoes of a mentor he emulates • BEARS (barriers to change, evidence of behavior and overcome barriers, resources needed for success, and strengths a client can draw upon). • Change, specific actions to accomplish the dream • Reflecting on the purpose of positive emotion • Raising awareness for the contribution of all valued areas of life for the overall well-being and the inclusion of these areas in the life priorities • Awareness of the “accept, change or leave principle” • Reflecting on past failures while attributing them externally • Reflecting on past success while attributing them internally • Reflecting on how problems were solved in the past • Values from a list: sort a deck of value cards into different piles according to how much each value printed on the card
Goal setting *f* = 10	Tools that aim to translate the desired positive end state (or Dream) of the client into actionable, achievable short- and long-term goals in different life domains (e.g., SMART goals).	• Applying CASIO brainstorming strategy • Using the Wheel of Life domains as a Brainstorming framework • Set SMART Goals and Objectives • Set two value informed, achievable, long- term, and short- term approach type goals in different life areas • Translating values into observable action steps • Mind maps to aid in goal setting and strength clarification • Developing a shared vision of the future
Strengths spotting *f* = 9	Refers to tools centered around active, deliberate, and constructive attempts to identify the manifested strengths of oneself or others (e.g., Strengths Camera).	• Strengths-Spotting / Listening for Strengths • Strengths-based interview • The Strengths Camera (Strengths ID): list a personal strength or value clockwise around the “lens” of the camera and then indicate how effectively they have been utilizing that strength, or implementing that value in the last 30 days • Strengths Map: Map how strengths can be used to achieve goals
Strengths utilization and development *f* = 9	Explore ways through which clients' strengths could be intentionally activated both at home and at work.	• Explore new ways to use strengths • Exploring ways to use strengths at work in life • Asking clients to use their top strengths in new ways for 1 week • Developing a plan for intentionally using strengths in the service of something or someone the person believes is worth fighting for
Creating a personal development plan *f* = 7	It is a strategic personal/ professional development map which translates strength development, developmental needs, and personal/professional goals into an actionable, implementable, and trackable operational strategy (e.g., The Appreciative Inquiry Framework).	• Help them develop a plan for reaching those goals, and then help him or her implement the plan and adapt it over time • Developing an action plan on how to build relationships and with whom • Developing a strategy for developing competencies • Identify specific competencies which need to develop in order to effectively manage the presented challenges, or highlighting the personal/professional goal the client wants to achieve, • Develop Action Plan to achieve goals • Determine resources needed • Determine how success will be measured and implement an action tracker
Building relationships and establishing rapport *f* = 6	Tools employed to develop a psychological contract between all stakeholders within the coaching process and to ensure a psychologically safe environment conducive to development (e.g., SOLER; Clarify expectations between coach/client).	• ARTS of communication: Ask first and listen, Respond with empathy, Teach your own perspective, Share decision making to achieve collaborative solutions • PEARLS: Partnership examples, Emotion/Empathy, Appreciation or apology, Respect, Legitimation, Support autonomy • Focus on what is going right and not what's going wrong • Establishing a psychological contract • Appreciative interviewing • Awareness on the 10, 40, 50% principle • Using curiosity to delve deeper into the client's world view • Positive introduction exercise
(Cognitive) reframing *f* = 6	Tools employed to cognitively reframe negative experiences in a positive manner or problems as opportunities (e.g., Telling stories as a survivor rather than a victim).	• Retell stories as survivors, not victims • Finding 15 positive things about a person who has done something hurtful • Reframe failures into learning opportunities • Appreciative interviewing • Pivoting: “the conscious act of turning attention from what the client does not want to what he wants” • Teaching the client how to reframe situations (look for the positive)
Resource activation *f* = 5	Guiding the client to rediscover and utilize existing but neglected personal, social, or environmental resources or energizing activities (e.g., reflecting upon past engaging activities).	• Exploring engaging activities • Identifying areas of flow and practicing flow activities • Identifying an expert friend who is doing well at handling the same challenges
Employing micro-skills (active listening) *f* = 5	Coaching communication skills employed that can aid clients to access their deepest thoughts, facilitate the development of self-insight and aids in creating an empowering environment conducive to change (e.g., Active listening).	• Framing questions in a positive, constructive manner (Positively Infused Language) • Active listening (attending behaviors, positive open questioning, paraphrasing and summarizing, noting and reflecting emotions, basic behavioral observation skills, nudging and influencing) • Solution-building conversations • Use affirmations to help the client more fully recognize and appreciate his or her effort, values, or achievement
Encouraging active skill development *f* = 4	Developmental interventions aimed at enhancing specific skills required to facilitate goal achievement.	• Social Competency Development (expressing affection and liking, general conversation skills, forgiving, apologizing, listening, assertiveness, affirming, and problem-solving) • Learning to enhance relaxation responses in the face of stress (e.g., Diaphragmatic breathing and mindfulness meditation) • Deliberate practice
Re-contracting *f* = 4	A technique used to reflect upon and evaluate the effectiveness of the coaching intervention at the end of the process in order to determine if further development is required/needed.	• Recontract relationship after completion of goals
Evaluating progress *f* = 3	Tools employed to evaluate the developmental- and goal achievement progress of the client throughout the coaching process (e.g., coaching logbook).	• Coaching log (record keeping) • Map development over time based on the Action Tracker • Measure life satisfaction on a monthly basis
Assessing personality, well-being, and work preferences *f* = 3	Psychometric instruments employed to assess the well-being, personality, and work preferences of the client (e.g., Baron EQ assessment).	• Diagnosing the position on the Languishing-Flourishing and Goal striving framework • Quality of Life Inventory • Baron Emotional Intelligence assessment • Positive Diagnosis: Identifying strengths, positive emotions and meaning, examining what the solution looks like • Belbin Leadership Roles • Myers-Briggs Type Indicator • 16 Factor Personality Index • Satisfaction with Life Scale
Managing difficult emotions *f* = 3	Techniques employed in managing destructive or negative emotions (e.g., practicing mindful awareness).	• Using Metaphors to clarify points • Employ expressive writing to draw out emotions • Employ loving and kindness Mediation • Practicing appreciative and expressive communications skills • Focus on solutions, not on the emotion • Practice mindful awareness • Employ strengths to buffer negative emotions • Develop stress management methods • Make lifestyle changes (healthy eating, exercise, etc.) • Parallel processing. Using the relationship between the coach and client to highlight what's going on in “real life”
Conducting competency-based assessments *f* = 3	Assessing specific competencies aligned to the role of the client (e.g., Developing and assessing the client against a positive capability matrix).	• Positive capability matrix • Simulations, job shadowing, and debriefing
Meaning making *f* = 1	A technique used to aid clients in crafting meaningful work experiences (e.g., job crafting)	• Job-crafting

### Selection Bias

We implemented three strategies to manage selection bias, enhance the credibility, conformability, and transparency of this systematic review. First, the initial search process, as outlined above, was independently conducted by two authors of this paper to ensure that no records were missed or excluded (29). Second, titles, abstracts and full papers were coded by a co-ordinating author in addition to one of the co-authors. Before each phase was completed, all four authors met to debate the reasons for the in- and exclusion of titles, abstracts, and final papers.

Moreover, to ensure inter-rater reliability, we utilized Cohen's kappa coefficient, which is a function of the relative observed agreement between raters (Pr_α_) from which the theoretical probability of agreement by chance (Pr_*e*_) is subtracted, and subsequently divided by the standardized probability of chance (35). The minimum recommended kappa level of 0.61 (36) was exceeded, which demonstrated substantial agreement between ratters (*k* = 0.87; *p* < 0.01).

### Classification of Techniques and Tools

Drawing on the PPCM (10) and based on the systematic literature review results, we aimed to identify the coaching phases and continuous processes in which the derived coaching tools/techniques are actively used. For this purpose, we engaged in a three-step approach.

First, all authors independently classified the derived coaching techniques to their respective coaching phases and/or continuous processes. Fleiss' Kappa was then calculated for every technique to evaluate the agreement between the four researchers' classifications. The agreement was evaluated based on Fleiss' recommendations for the interpretation of Kappa, i.e., 0.75–1.00 indicating an excellent agreement, 0.40–0.74 a fair to good agreement, and values below 0.40 a poor agreement (37).

In the next step, the opinion of six independent experts in the field of positive coaching psychology was sought. Based on their scientific and practical experience, the experts were asked to link the derived coaching techniques to the phases or processes of the PPCM (10) in which the respective techniques are most actively used. For this purpose, the experts were provided with (a) descriptions of the techniques and respective tools as well as (b) a link to the paper of Van Zyl et al. (10) to ensure that the experts have a clear understanding of the model as well as derived technique Finally, Fleiss' Kappa based on only the experts' classification, was calculated for each coaching technique.

Once all results were obtained, a meeting was held in which all authors discussed the experts' and authors' classifications with the aim of deriving at a final classification. The following criteria was used to finalize the classification: (a) at least seven persons agreed on a respective classification, among them (b) at least half of the research team (*n* = 2) and (c) half of the experts (*n* = 3). Where disagreement was still present during the final integrated classification phase, such was noted and the frequency of total classifications were then used as the final guiding principle for classification.

## Results

### Common Techniques and Tools Used in Positive Psychological Coaching

The data obtained *via* the systematic literature review was processed using thematic content analyses. From the 24 articles, 117 different coaching tools could be extracted. Based on these tools, we derived 18 overarching coaching techniques applied by coaches during a positive psychological coaching process. Table 1 provides a descriptive overview of the techniques, the associated tools employed and their frequency of occurrence.

*Providing Self-Administered Intentional Activities*. The majority of the examined articles (*f* = 15) endorsed the provision of brief evidence-based positive psychological self-administered intentional activities. They represent a means of supporting clients in deepening insights as well as encouraging practice outside the coaching sessions. Frisch points out that such “homework” may foster “the effectiveness of in-session interventions by having clients think about and implement in-session ideas and techniques between sessions and after coaching […] is over.” [(15), p. 217]. Thus, these activities may ensure the continuous transfer of insights into the clients' everyday life. Coaches can choose from a wide range of exercises depending on the goals that should be pursued. For example, Kauffman (38) proposes a gratitude visit to enhance positive emotions about the past. Activities such as performing acts of kindness and meditating on positive feelings toward self and others were shown to increase well-being, specifically individuals' happiness (39).

*Strengths-Focused Psychometric Assessments*. Another highly recommended technique (*f* = 15) relates to the assessment of strengths by using simulation exercises or established inventories, e.g., the VIA signature Strengths (40), the Realise2 (41), or the Gallup Clifton Strengths Finder 2.0 (42). These assessments are applied to systematically identify strengths and show potential developmental needs (41). Moreover, the application of standardized inventories facilitates the examination of a client's strengths from an objective viewpoint. In this way, also unconscious strengths can be brought to light (9).

*Guided Self-Reflection*. Fourteen out of 24 articles suggested tools associated with gaining insights under supervision. Compared to self-administered activities, the technique *Guided Self-Reflection* encompasses tools that are implemented within coaching sessions to aid the client in systematically discovering his/her potential, strengths, and solutions to manifested problems through active involvement and guidance from the coach. Here as well, a wide range of specific tools were suggested depending on the objective. Aiming at providing tools to enhance the experience of positive emotions, Anstiss and Passmore (43) describe that coaches may firstly initiate a talk about the importance of positive emotions followed by questions about the client's current experiences and further joint exploration of possibilities to stimulate the experience of positive emotions in the future. Furthermore, some of these tools give an orientation how coaches may structure the elaboration of different topics [e.g., by following the BEARS approach; (44)].

*Goal setting*. As the third most frequently occurring technique, 10 articles mentioned the deployment of specific goal setting techniques to facilitate personal and professional development in a structured and systematic way. The focus here lies on tools that aid the client in setting achievable short- and long-term goals in different life domains to achieve a desired state (9, 38). Most articles referred to the use of the popular SMART (specific, measurable, attainable, realistic and time-bound) goals technique (9, 44, 45) or employing the Wheel of Life domains framework to brainstorm ideas around the specific needs of the client (38). This aided in crafting a shared vision of the future which guides the coaching process.

*Strengths Spotting*. Strength-spotting (*f* = 9) relates to an active, deliberate and constructive process initiated by either a coach to identify the manifested strengths of the client, or by a client to identify her/his own strengths or those of others. These techniques and the respective tools are used to provide the client with the strengths-vocabulary, -diagnostic framework and criteria needed to identify the strengths of oneself or others. These tools are more qualitative in nature. For example, Kauffman proposes a positive introduction exercise where based on events from the prior week, clients are asked to describe themselves at their very best and thus learn to focus specifically on their strengths (38).

*Strengths Utilization and Development*. With an equal frequency (*f* = 9), articles also emphasized tools focusing on utilizing and developing the clients' strengths. The respective tools aim at exploring ways through which the strengths could be intentionally activated both at home and at work. Here the focus is on deliberate strengths-use to attain goals or to address developmental areas. Additionally, clients may also search for ways to use their strengths in a new way (46).

*Creating a Personal Development Plan*. Seven out of 24 articles referred to the construction of a personal development plan (PDP). The PDP refers to a strategic personal/professional development map that translates strength development, developmental needs, and personal/professional goals in to an actionable, implementable operational strategy (26). The plan highlights the clients' ideal vision of their desired future, areas of strength, development, and specific competencies that clients present with. This PDP also captures the specific short- medium- and long-term goals of the client, which are broken down into clear action steps, deadlines, means to track progress and clarifies the support/resources needed to aid in goal achievement (9). Furthermore, the PDP highlights the resources, support and developmental interventions needed to aid the client to bridge the current vs. desired state gap (5). A popular framework suggested was Appreciative Inquiry as it provides a structured, “positive psychological approach to frame solutions and develop action plans” (5).

*Building relationships and establishing rapport. Six* articles suggested tools to clarify expectations and set up rapport between coach and client. These tools are used to develop a psychological contract between the stakeholders and a psychologically safe environment conducive to development (9). For example, several articles recommended the SOLER technique by Egan (24) “facing the coachee squarely, displaying body language that can be considered open and inviting, leaning toward the coachee to display interest, ensuring eye-contact is made and relaxing as to ensure a comforting climate is created for the coaching process” [(9), p. 284]. Van Zyl and Stander (26) sum up other tools that may build the basis of a good contact between coach and client, e.g., creating a calm and trusting environment, clarifying expectations, presenting a genuine unconditional positive regard.

*(Cognitive) Reframing*. Just as frequently, six articles mention tools that could be used to cognitively reframe negative experiences in a positive manner or problems as opportunities (5, 9, 38). For this purpose, clients can be requested to re-tell their negative stories from a survivor rather than a victim perspective (5, 9, 26). Kauffman (38) describes an optimism-building exercise where clients are asked to reflect upon their lives and specific times when they did not succeed, or when their plans were ruined. In the next step, clients have to reveal what good things resulted from these situations.

*Resource Activation*. Resource Activation was mentioned by five articles as a technique aimed at guiding the client to rediscover and utilize existing but neglected personal, social, or environmental resources, e.g., social support networks, mentors, etc. (9), or energizing activities (43). In this regard, Anstiss and Passmore (43) suggest “exploring with the client the kind of activities he or she currently finds engaging, the things she or he used to find engaging but have stopped doing, and the activities she or he might wish to do more of in the future.” (p. 244).

*Employing Micro-Skills (Active Listening)*. Five articles mentioned the use of micro-skills by coaches in order to facilitate meaningful conversations with clients. This technique represents a fundamental coaching communication skill that can aid clients to access their deepest thoughts, facilitate the development of self-insight and aids in creating an empowering environment conducive to change (5). For example, Van Zyl and colleagues propose active listening and the use of positively infused language (5, 26). This includes attending behaviors, positive open-questioning, paraphrasing and summarizing, noting and reflecting emotions, basic behavioral observation skills, nudging, and influencing (5). Additionally, Anstiss and Passmore (43) endorse the use of affirmations “to help the client more fully recognize and appreciate his or her effort, values, or achievement” (p. 245).

*Encouraging Active Skill Development*. Besides strengths development, several articles (*f* = 4) also endorse the active development of skills in positive psychological coaching. This refers to developmental interventions aimed at enhancing specific skills, competencies or capabilities required to facilitate effective and efficient goal achievement (47). For example, Anstiss and Passmore (43) propose developing social competencies to support positive relationships. Furthermore, Kauffman et al. (47) emphasize the value of methods to reduce physiological activation in the face of stress, e.g., mindfulness meditation.

*Re-Contracting*. With an equal frequency, four articles referred to re-contracting as a summative technique used to reflect upon and evaluate the coaching process to determine whether further development is required. Van Zyl and Stander (26) point out that initiating a new coaching process may be appropriate if expectations have not been met at the end of a current coaching process, and thus, the client expresses the need for further development.

*Evaluating Progress*. In three articles, the authors mention tools used to evaluate clients' developmental- and goal achievement progress in the coaching process. For example, Stander (9) highlights the application of a coaching logbook to revisit expectations at regular intervals.

*Assessing Personality, Well-being, and Work Preferences*. Apart from assessing strengths, *three* articles suggested psychometric tests to be employed to assess the clients' personality, well-being, and work preferences. The results are valuable to develop a holistic picture of the client and to ensure contextual alignment to the environment in which he/she functions. Van Zyl et al. (5) mention the Belbin teamwork test (48) as an example to assess preferred roles when working in teams.

*Managing Difficult Emotions*. While most articles focused on strengthening positive emotions and well-being, *three* articles discussed additional tools that can be applied to deal with clients' difficult emotions, e.g., expressive writing or practicing mindful awareness (49).

*Conducting Competency-Based Assessments*. Similar to strengths-based assessments, three articles emphasized the need to assess specific work-related competencies. The authors suggested the development a positive capability matrix comprised out of strengths-based competencies (e.g., strategic visioning), experiences (e.g., career accolades), abilities (e.g., learning potential), and values (e.g., authenticity) (5, 9, 26). Competencies and experiences act as indicators of individual performance, whereas individual potential is estimated *via* ability and values. Clients can be assessed against these competencies through, for example, fit-for-purpose simulations, job shadowing, and debriefing.

*Meaning Making*. One article discussed the value of tools aiding clients in crafting meaningful work experiences (5). This may include job-crafting activities.

### Classification of Positive Psychological Tools and Techniques Into the PPC Model

The second objective of this paper was to classify the identified psychological tools and techniques into a known positive psychological coaching framework: the PPCM (10). An iterative heuristic classification process was employed with three steps: First, researchers independently classified the coaching themes into their respective categories. Second, the same was done by six independent experts and third, the results were combined to derive at a final classification.

### Step 1: Researcher Classifications

The researchers individually classified the 18 coaching themes into their respective coaching phases/ processes. The results are depicted in Table 2. Based on Fleiss' recommendations for the interpretation of Kappa, we found an excellent agreement for five coaching techniques, i.e., encouraging active skill development; assessing personality, well-being, and work preferences; building relationships and establishing rapport; (cognitive) reframing as well as employing micro-skills. The research team members' classification showed a fair to good agreement on nine techniques, e.g., evaluating progress and meaning-making. However, a poor level of agreement was established for competency-based assessment, managing difficult emotions, creating a personal development plan, and strengths spotting.

**Table 2:** Authors' and experts' classification of the coaching techniques with regard to their predominant use along the phases and processes of the PPCM (10).

**Technique**	**Rater**	**Phase 1: creating the relationship**	**Phase 2: strengths profiling and feedback**	**Phase 3: developing an ideal vision**	**Phase 4: goal setting, strategizing, and execution-based on strengths**	**Phase 5: concluding relationship and re-contracting**	**Continuous process 1: learning transfer**	**Continuous process 2: action tracking and continuous evaluation**	**Continuous process 3: empowerment**	**Fleiss' Kappa**
1. Encouraging active skill development	Authors				1, 2, 3, 4		1, 2, 3, 4		1	0.85
	Experts		C		B, D, E, F			E	B	0.23
2. Psychometric assessments focused at personality, well-being, and work preferences	Authors		1, 2, 3, 4			2, 3, 4		1, 2, 3, 4		0.86
	Experts	E	A, B, C, D, E, F	F				B, D, E		0.55
3. Building relationships and establishing rapport	Authors	1, 2, 3, 4			1	1, 2, 3, 4				0.85
	Experts	A, B, C, D, E, F				C, E			B	0.64
4. (Cognitive) reframing	Authors		1, 2, 3, 4	2, 3, 4	1, 2, 3, 4		1, 2, 3, 4	1		0.75
	Experts		C, E	A, B, F	C, F		B		D, E	0.04
5. Conducting competency-based assessments	Authors	1	1, 2, 3, 4	1	1	2, 3, 4		1, 4		0.29
	Experts	B	A, C, D, E, F	F	F		F	B, E, F	D	0.18
6. Evaluating progress	Authors				1, 4	2, 3, 4		1, 2, 3, 4		0.64
	Experts				B	A, B, F	A, E, F	A, B, C, D, E, F	F	0.44
7. Goal setting	Authors			1, 4	1, 2, 3, 4	1, 2, 3, 4		1, 4		0.64
	Experts	B		A, C	C, D, E, F	F	E		B	0.09
8. Guided self-reflection	Authors	2, 3, 4	1, 2, 3, 4	1, 2, 3, 4	1, 2, 3, 4	2, 3, 4		1	1, 2, 3, 4	0.54
	Experts	B	A, C, D, E, F	D, F	F	B	E	E	B, E	0.07
9. Managing difficult emotions	Authors	2, 3	2, 3	2, 3, 4	1, 2, 3	2, 3, 4	1, 2, 3, 4	2, 3	1, 2, 3, 4	−0.08
	Experts	B, C, E	C, E	C, F	B, C, E, F	F	A, F	F	B, D, E	−0.07
10. Meaning making	Authors			1, 4	1, 2, 3, 4		1, 2, 4		1, 3, 4	0.56
	Experts		C	A, B, F	C, E, F		E	E	D, E	0.03
11. Employing micro-skills (active listening)	Authors	1, 2, 3, 4	1, 2, 3, 4	1, 2, 3, 4	1, 2, 3, 4	1, 2, 3, 4	1, 2, 3, 4	1, 2, 3, 4	1, 2, 3, 4	1.00
	Experts	C, D, E, F	C, D, E, F	C, D, E, F	A, B, C, D, F	C, D, F	C, F	C, D, F	A, C, D, E, F	−0.07
12. Creating a personal development plan	Authors		1	1, 4	1, 2, 3, 4		1	1, 4	1	0.22
	Experts	B	B		C, D, F	A, F		A, B, E		0.09
13. Providing self-administered intentional activities	Authors		2, 3, 4	2, 3, 4	1, 2, 3, 4	2, 3, 4	1, 2, 3, 4		1, 2, 4	0.46
	Experts			F	A, B, F	F	C, D, E		B	0.10
14. Re-contracting	Authors					1, 2, 3, 4		1, 4		0.73
	Experts					A, B, C, D, E, F		E		0.83
15. Resource activation	Authors			1, 4	1, 2, 3, 4		1, 2, 3, 4		1, 2, 4	0.70
	Experts		B, C, E, F	F	A, B, F	B	C, E		B, C, D, E	0.17
16. Strengths spotting	Authors	1, 2, 3, 4	1, 2, 3, 4	1, 2, 3, 4	1, 2, 3	2, 3	1, 2, 3, 4	2, 3	1, 2, 3, 4	0.13
	Experts		A, B, C, D, E, F				E	B		0.70
17. Strengths utilization and development	Authors		1	1, 4	1, 2, 3, 4		1, 2, 3, 4		1, 2, 4	0.58
	Experts		F, B, C		A, C, D, E, F	F	E	F, B		0.29
18. Strengths-focused psychometric assessments	Authors		1, 2, 3, 4			2, 3, 4		1, 4	1	0.52
	Experts	B	A, B, C, D, E, F			E	C	B, C, E		0.47

*Letters A–E refer to the single experts' classification. Numbers 1–4 represent the classification of the respective authors*.

### Step 2: Expert Classifications

In the second step, six experts provided their own independent classification. Table 2 displays the experts' results together with the Fleiss' Kappa for each coaching technique (only based on the experts' classification). Only for one technique, i.e., re-contracting, experts showed excellent agreement. A “fair” to “good” level agreement was found for five coaching techniques, whereas for 11 techniques, the experts' opinions diverged widely, resulting in a poor agreement.

### Step 3: Final Classification

Third, the results stemming from Step 1 and 2 were integrated into a common classification scheme based on the predefined integration criteria. The results of the integration process are depicted in Table 3. Ten of the coaching techniques were applicable to at least two of the coaching phases. However, the use of micro-skills was deemed to be relevant to each phase of the coaching process. Only seven techniques (Building relationships and establishing rapport, strength spotting, strength utilization and development, goal setting, meaning-making, creating a personal development plan, and re-contracting) were specifically deployed in a single coaching phase.

**Table 3:** Focal application of coaching techniques during the positive psychological coaching process.

**Technique**	**Phase 1: creating the relationship**	**Phase 2: strengths profiling and feedback**	**Phase 3: developing an ideal vision**	**Phase 4: goal setting, strategizing, and execution based on strengths**	**Phase 5: concluding relationship and re-contracting**	**Continuous process 1: learning transfer**	**Continuous process 2: action tracking and continuous evaluation**	**Continuous process 3: empowerment**
1. Encouraging active skill development				**X**		**X**		
2. Psychometric assessments focused at personality, well-being and work preferences		**X**					**X**	
3. Building relationships and establishing rapport	**X**							
4. (Cognitive) reframing		**X**						**X**
5. Conducting competency-based assessments		**X**					**X**	
6. Evaluating progress					**X**		**X**	
7. Goal setting				**X**				
8. Guided self-reflection		**X**						**X**
9. Managing difficult emotions				**X**				**X**
10. Meaning making				**X**				
11. Employing micro-skills (active listening)	**X**	**X**	**X**	**X**	**X**	**X**	**X**	**X**
12. Creating a personal development plan				**X**				
13. Providing self-administered intentional activities				**X**		**X**		
14. Re-contracting					**X**			
15. Resource activation				**X**		**X**		**X**
16. Strengths spotting		**X**						
17. Strengths utilization and development				**X**				
18. Strengths-focused psychometric assessments		**X**					**X**	

## Discussion

The purpose of this paper was to identify which positive psychology coaching tools and techniques positive psychological coaching researchers employ and how such can be classified into the various phases of Van Zyl et al.'s PPCM (10). *First*, a systematic review was used to determine which coaching tools and techniques stem from the positive psychological coaching literature. The results showed that positive psychological coaches employ 18 types of PPC techniques and 117 different coaching tools to aid clients in their personal and professional development journeys. Each set of these techniques is comprised of various evidence-based tools or strategies ranging from psychometric assessments to SMART goal setting and job-crafting. Second, an iterative heuristic classification process was employed to systematically associate the tools and techniques into the various phases of Van Zyl et al.'s PPCM (10). The results showed that most tools and techniques should be employed in at least two phases/ continuous processes; however, the results also highlighted significant differences between professional coaches in how tools/techniques should be classified.

### Positive Psychological Coaching Tools and Techniques

The results showed that positive psychological coaches have a wide array of resources at their disposal to aid clients to achieve their personal- or professional goals. It would seem as though the most popularly suggested tools or techniques pertain to the use of *psychometric instruments aimed at identifying clients psychological or signature strengths*. The popularity of such could be attributable to the fact that positive psychology is considered the scientific study of psychological strengths and that strengths-based assessment forms a large part of this paradigm (50). As PPC aims to identify and utilize psychological strengths, assessment of such is a natural step in the process (10). Therefore, coaches need to have access to and be competent in using a wide array of strengths-based psychometric assessment measures to scientifically determine the manifested or unconscious strengths of a client (51). These assessment tools could either be aimed at measuring inherent psychological (or character) strengths [e.g., VIA Signature Strengths Inventory; (52)] or behavioral strengths (or competencies) through the Clifton Strengths Finder 2.0 (53). Strengths-Based psychometric tools provide a means to assess these underlying strengths and give way to a conversation around the function and purpose of these strengths in clients' lives (54). This, in itself, leads to a heightened awareness of one's strengths when it can be used and could lead to reframing events from a strengths-based perspective (26).

*Strengths Spotting* was also identified as an important (qualitative) tool positive coaches can use to identify strengths. It differs from the use of psychometric instruments in the sense that the coach facilitates an active and constructive process/conversation to help identify the client's strengths qualitatively (without the use of a psychometric instrument). The advantage of this technique is identifying strengths taking place in the context of the client's work and social environment. This ensures that the identified strengths are more relatable and contextualized within the clients' environment. Relating to the strengths will enable the client to utilize and reinforce the strengths. For example, Kauffman (38) propose an exercise to identify strengths based on behavior and experiences from the prior week. Although formal approaches (psychometric instruments, self-administered instruments) are, arguably, the most common methods of identifying strengths, there are also more informal methods, such as strengths spotting. Fouracres and Van Nieuwerburgh (55) postulate that coaching theory itself does not imply that coaches require objective measures to ensure successful coaching outcomes. They suggest that self-identification, as an alternative to objective psychometrics, allows clients to identify their own strengths, which in turn increases the active use thereof in clients' daily lives.

Strengths spotting is an open-ended method of observing behavioral cues with the purpose to identify a client's strengths. The advantage of more open-ended or qualitative approaches is that the language and construction of the strengths are grounded firmly in the client's lived experience; ensuring authenticity and ownership (56). Strengths spotting involves the careful, intentional observation of strengths within the stories, interactions, and behaviors of others or oneself. It involves the labeling of observed strength(s) and offers a rationale for how it was expressed (57). Facilitating clients to identify their own strengths could allow the client space to engage in both self-insight and self-reflection, permitting the observation or strength to transfer from the sub-conscious into visibility and motivate conscious actions (55). When using strengths spotting, it is essential to facilitate a process *with* the client to determine the extent to which they, themselves, identify with a particular strength (56). Gaining awareness of other's strengths is dependent on how openly they display strengths, and one's own ability to observe strengths in others (58). This technique will expect the coach to be sensitive and continuously practice observing strengths in everyday interactions. Furthermore, the coach must strive to develop clients' competence in self-insight and self-reflection to the extent that it becomes a habit to observe strengths. Strengths spotting and strengths utilization and development is at the heart of a PPS approach.

Equally as prevalent, the results showed that authors suggest using evidence-based positive psychological *self-administered intentional activities* (e.g., Gratitude Visit) to aid clients in enhancing their well-being or to practice strengths in a validated manner. These evidence-based practices refer to tools developed to enhance specific positive states (e.g., happiness), traits (e.g., hope) or behaviors (e.g., deliberate practice) (59). These positive psychological tools provide structured guidelines for enhancing a particular state/trait/behavior and do not require the presence or support of the coaching practitioner (56). These can be classified into three broad categories of tools: First, *cognitive tools* which aim to change how a client thinks about him/herself, a given event or the future such as visualizing an ideal future self, or self-monitoring (positive journaling) (60). Second, *behavioral tools* which require clients to action or show a particular behavior such as looking for ways to use strengths in a new way or performing random acts of kindness (59). Finally*, emotional tools* which clients can use to relive positive experiences from the past (e.g., gratitude visit), extending positive experiences in the present (e.g., savoring life's joys) or to anticipate positive experience in the future (e.g., practicing optimism) (52). These tools should not be applied in a prescriptive manner but rather be strongly aligned to clients' strengths and their goals for them to be effective (19).

A further prominent factor emanating from the literature was *Guided Self-Reflection*. These techniques relate to strategies coaches can employ to facilitate clients in discovering their own hidden potential (e.g., reflecting on successes), re-enforce strengths use (e.g., appreciative questioning), or to generate solutions to problems (e.g., sense checking or reflecting on how similar problems were solved in the past) during a given coaching session. Although guided self-reflection is not own to the positive psychological paradigm (61), it is applied uniquely within the PPC process. Here, clients are guided to focus on or look for positive experiences or outcomes of a given event, where the specific emphasis is placed on the role and use of psychological strengths and positive emotions (62). In traditional coaching frameworks, clients are guided to identify the underlying causes of poor performance (or other problems) and generate solutions to compensate for such (46). From the positive perspective, clients are guided to explore the positive, focus on what already works well and determine ways and means to optimize such (63). Therefore, the focus is firmly placed on the tools required to facilitate clients to become more mindfully aware of the positive aspects of their lives and to reinforce this mindset.

*Goal Setting*, as a technique to clarify the needs of a client in order to structure the developmental process, was identified as one of the more frequently used techniques reported in the PPC literature. This technique is part of most, if not all, coaching models and approaches (10). Goal setting forms an integral part of any training and development process or intervention. Achieving these goals will determine the success of the coaching process. Locke and Latham's seminal work on the Goal-setting theory is based on the underlying assumption that conscious human behavior is purposeful and regulated by the individual's goals, given the person has the requisite ability, goal-directedness will motivate and drive the actions of people (64). Within a PPC approach, Cheavens et al. (65) postulate that from hope theory, goals create the context for developing specific pathways and agency thoughts and serves as a means for people to use feedback from goal outcomes to inform their future actions. They furthermore state that goals perceived as important by the client will ensure that they are more intrinsically motivated to implement strategies to facilitate goal achievement. Latham (66) suggests that setting specific goals will increase the likelihood that people will respond positively to the feedback they receive in goal progression. Yalom (67) believes that concrete, attainable goals defined by the client, will increase their sense of responsibility for taking ownership of their own development. Various tools to aid in setting clear and concrete goals were identified in this study. Most articles referred to the use of the famous SMART (specific, measurable, attainable, realistic and time-bound) goals (9, 44, 45) that can be formulated to achieve short- or long-term goals in different life domains (6, 38). This technique will set the direction and action plans of the PPC process. In this respect, goal setting from the PPC differs from traditional coaching in the sense that it is not only training needs-driven, but can also focus on optimizing the use of strengths (63).

*Strengths Utilization and Development* is aimed at exploring ways through which strengths could be intentionally activated both at home and work. The success of a PPC approach rest strongly on the optimization of identified strengths. Coaching interventions should therefore not only involve the identification of strengths, but also focus on using strengths in an innovative way or as a means to achieve a goal (7, 38, 46). Identified strengths can be cultivated through practice and developing related knowledge and skills so that they can ultimately be productively applied (58). Strengths can be seen as “those (trait-level) personality characteristics that, when activated (state-level), are associated with the optimal functioning of a particular person” [(58), p. 3]. For organizations to benefit from this strengths-based focus, Biswas-Diener et al. (68) believed that buy-in to this approach should be established at all levels of an organization. Specifically, organizations should construct strengths-based capability models that aims to identify, explore, develop and celebrate the strengths of individuals and teams. Establishing a strengths-based climate may increase the ability organizations to recognize and appreciate the function of strengths in the professional development journeys of individuals and the function of strengths in team contexts (58). Creating such a culture will be supportive of a PPC approach and aid in fast tracking the development of employees.

Employees need to be encouraged to explore or optimize their strengths in different work-related contexts through structured strengths-based utilization and development approaches. One example of such is appreciative inquiry (AI), where the focus is on what people or teams do exceptionally well. When applied in the coaching process, this facilitated conversation approach emphasizes confirming language, exploring past successes, identifying strengths and resources, and transforming abstract values into concrete goals and behavioral efforts to facilitate healthy work-related changes (69, 70). This leads to valuable individual- (e.g., mental health), team- (e.g., team flow), and organizational (e.g., performance) outcomes (12).

Further, our results showed that some tools and techniques were less prevalent and, therefore, seem to not be specific to the positive psychological coaching approach but rather relate to generic coaching practices. These include tools and techniques relating to: the creation of a personal development plan, building relationships and establishing rapport, cognitive reframing, resource activation, employing micro-skills, encouraging active skills development, re-contracting the coaching relationship, assessing other personal characteristics (personality, well-being, and work preferences), ways to manage difficult emotions, conducting competency-based assessments, and meaning-making activities. These tools and techniques are generic in nature and foundational approaches in all coaching models, processes, or interventions (12).

### Operationalising the Positive Psychological Coaching Model With Tools and Techniques

The final component of this paper was to determine how the identified tools and techniques could be classified into a known PPC framework to operationalize the model further. This in turn would aid practitioners in selecting the right tool/technique necessary to address the needs of a client at the right time (71). Through an iterative heuristic classification process, it was found that the majority of the coaching tools and techniques could be classified or “used” in at least two of the PPC model's phases/processes. The results showed that there was poor agreement in how 11 of the 18 techniques should be classified into Van Zyl et al.'s PPCM. Given that the coaching interventions do not follow a linear path, these coaching tools and techniques could therefore be used in multiple phases and at different times during the coaching relationship (22). Our results therefore highlight the fluidity in the use of these PPC tools/techniques throughout the coaching process. Table 3, therefore provides a visual representation of the final classification framework and highlights how each tool or technique relates to a given phase/process of the PPCM.

The results showed that tools or techniques associated with *building relationships and establishing rapport* as well as the use of *micro-skill*s are associated with Phase 1 (*Creating the Relationship*) of the PPC model. This phase is primarily focused on establishing a positive relationship with the client, as the success of the process is fundamentally dependent thereon (19). These tools and techniques would aid the coach to ensure that he/she understands the nature of the clients' work and those factors that matter most. Through employing micro-skills, the coach ensures that (a) a client feels “heard” (i.e. it communicates empathy and understanding), (b) a psychologically safe environment is created, and (c) fosters a positive relationship that is conducive to change (72). These strategies, therefore, place the relationship first and aids in not only establishing rapport but also facilitates in building a positive working relationship conducive to change.

Phase 2 (*Strengths Profiling and Feedback*) of the PPC model relates to the means through which clients psychological or behavioral strengths are assessed and how feedback is provided. This phase is strongly associated with strengths-based assessment, and it incorporates a wide variety of techniques to identify strengths. The results showed that both *strengths-focused and general psychometric assessments, competency-based assessments, strength spotting initiatives, micro-skills use, guided self-reflection, and cognitive reframing* could be employed in this phase. The assessment is supported by processes to reconfigure possibilities and look for opportunities (73). The client should be aware of his/her strengths and develop self-insight as to how these strengths could be used to foster personal development and achieve goals (8, 51).

In Phase 3, (*Developing an Ideal Vision)* the client develops a clear picture of the perfect version of him/herself in the future. The results indicate that *micro-skill*s is associated with this phase. It entails communication skills that enable clients to access their deepest thoughts, facilitate self-insight development, and determine an ideal state as an outcome of coaching. Carkhuff (61) in his well-known work on “the art of helping” emphasizes the importance of attending (physical, active listening, and observing), responding (empathy, respect, and warmth), and personalizing (helping the client to understand where he/she wants to be) as crucial skills in interacting with the client. Instead of directing the discussion, the coach should encourage the client to develop self-insight (74). Furthermore, these authors recommended that coaches (themselves) should also engage in self-reflection to enhance their own self-insight and well-being. Passmore and Oades (73) support a positive case conceptualization approach where possible preferred situations are identified with the client. Instead of diagnosing deficits, possibilities and opportunities should be explored and formulated to direct the coaching process going forward.

Phase 4 (*Realistic Goal Setting, Strategizing and Execution)*, is a core function within the coaching process and its therefore not surprising that a large number of tools and techniques seemed to apply to this phase. The coach must facilitate a process to set specific, measurable, attainable, realistic, and time-bound goals aligned to the client's strengths and ideal vision. Grant and O'Connor (74) confirm the importance of understanding why the goal is set and that the coach and client agree with the reason for achieving the specific goal. These goals need to be translated into a clear personal development plan, with specific implementable actions. A wide array of techniques could be applied within this phase of the coaching process, *including managing difficult emotions, meaning-making, micro-skills, self-administrated activities, resource activation*. Within the PPC approach, the core focus in this phase will be on and strengths development. The coach must assist the client in appreciating the power and opportunities that his/her strengths provide the client (42). According to Passmore and Oades (75), the client will perform, feel and function better when using their strengths. These can all be outcomes of the coaching process. The coach needs to encourage the client to identify potential personal resources and coaching themes as these will direct the coaching process (76).

The results further showed that tools or techniques associated with *evaluating process, re-contracting*, as well as the use of *micro-skill*s, are primarily associated with Phase 5 (*Concluding Relationships and Re-contracting*). Given that this phase signifies the end of the current coaching trajectory, appropriate tools and techniques need to be deployed to either finalize the coaching process or prepare the client for another developmental journey. Evaluative and reflective practices are therefore important. Passmore (51) briefly describes three evaluation options: First, using psychometric instruments or a 360-competence assessment in a pre- and post-measurement. Second to compare data, for example absenteeism, before and after coaching and lastly assessing overall performance like sales made. It will be essential to identify and control all contamination factors. The ideal will be to apply a five-level evaluation with a return on investment as the ultimate measure as proposed by Phillips et al. (77). Towards the end of the coaching process, the client must be prepared to conclude or re-negotiate the relationship (26). In some cases, the coach and client will jointly decide to continue with the coaching process. If goals were achieved, new goals could be set, and the coaching relationship continues. In re-contracting, it is once again essential to get mutual agreement on goals, roles and expectations. Clarity and positive feelings of shared purpose will increase trust in the coaching intervention (78).

The sequential phases of the PPCM are supported by three dynamic or “continuous processes” that strengthens the interaction between the different phases. These three continuous processes apply to all the phases of the coaching model, build on, and are supported by each other (10). In *Continuous Process 1* (*Learning Transfer*), learning from the coaching process should be transferred to the work environment while the client takes ownership of the learning process. Cook (79) states that organizations have high expectations for coaching. One of these expectations is that the process will enable learning in the workplace. Four techniques were prominent in this continuous process, *active skill development, micro-skills, self-administered intentional activities and resource activation*. Taking into consideration that there is an interaction between phases, one should expect a variety of overlap in tools and techniques used in this phase/process. The purpose of these techniques is to guide the client to rediscover and utilize existing personal, social, or environmental resources and align it to their own as well as the company's advantage. In addition, the coach can provide “homework” to aid the client in developing competence in adherence to the coaching process (60). Clients need to be empowered to introduce newly learned practices or skills in the workplace and be made aware that failures should be seen as a learning opportunity (9, 38).

*Continuous Process 2* (*Action Tracking and Continuous Evaluation*) aims to track the effectiveness of the intervention in both goal achievement and an increase in well-being. Tracing the developmental process ensures that the coaching intervention supports the client to achieve his/her goals and to intervene if evidence suggests that the client is not on track with his/her goal achievement (26). Four techniques were prominent in this process, namely *psychometric assessments, micro-skills, competency, and strengths-focused assessments*. Although the PPCM has a definite start and end date, within an ambiguous business environment, the development process in itself is not linear (10). Continuous changes in the client's roles, or the demands of the business, could result in the reformulation or reprioritization of goals. Should changes or demands in the developmental process occur, the coach and client need to re-prioritize goals to ensure goal achievement is still on target (9). Continuous assessment (*psychometric, competency, and strengths-focused)* enables the coach to assess the client's progress. Using micro-skills are imperative to facilitate the identification of goals and to help the client formulate concrete plans to achieve these goals (61). The coaching process should therefore incorporate active elements to monitor and evaluate the development of the client to ensure that active steps are taken toward goal achievement (46).

*Continuous Process 3* (*Empowerment, Reframing, and Reinforcement*) aims to aid the client to experience a sense of control over initiating and regulating behavior to make a difference in their context. The focus is on reframing challenges as opportunities and to find the positive in negative experiences. This continuous process applies to each of the five chronological phases and supports both learning transfer and the evaluation process. Five techniques (*reframing, guided self-reflection, managing difficult emotions, and resource activation*) were identified as essential in this phase. Here, positive confirmations and positively infused questions should be used to reinforce clients' faith in their own strengths (46, 80), challenges should be reframed as opportunities, clients' needs to be assisted to internalize strengths as personal resource, and client should be facilitated to move away from a victim to a survivor-oriented mindset (5). The coach should strive to empower the client to take ownership of his/her personal development and reduce his/her dependency on external resources (80). Noble et al. (81) describe empowerment as the linchpin of the strengths coaching model. They value a coach that believes in the client's ability to cope and change in positive ways.

### Limitations and Recommendations

Although various strategies were implemented to enhance the relevance and rigor of the current study, a number of limitations in terms of the research design and generalizability is apparent. First, the current review focused solely upon theoretical papers aligned to the original PPCM and not empirical studies investigating the effectiveness of PPC as an intervention framework. In intervention studies, authors are encouraged to fully describe the intervention protocol, including specific tools and techniques used (27). As such, empirical papers which are aligned to the PPC paradigm might become interesting sources of information relating to tools and techniques in the future. Second, only peer reviewed, academic manuscripts or chapters were included in the review, therefore excluding gray literature and popular psychology/management books. Although these sources are not aligned to the scientific method nor exposed to the rigor of peer-review, they do usually provide more practice orientated guidelines, strategies, tools, and techniques than the academic literature. Therefore, a number of PPC tools and techniques may have been excluded. Third, the classification process involved only a single round of iterative heuristics. Although the methodology employed was rigorous, a number of additional iterations of review, classification and refinement could have increased the inter-rater reliability and aided in providing more evidence for the final classification (especially on the factors where low inter-rater reliability was established). Fourth, although this systematic literature review and classification provides an overview of various positive psychological tools and techniques, it does not provide evidence as to the effectiveness or usefulness thereof. Therefore, it is not known to what extent each specific tool or technique contributes to the effectiveness of the overall positive psychological coaching process. It is suggested that future researchers embark upon a meta-analysis in order to determine the specific effect each coaching tool or technique has on desired positive psychological outcomes during the coaching process. Finally, the identified experts were only sent brief instructions, coupled with a very short description of each phase. Therefore, each rater may have not fully understood each component or interpreted it form his/her own paradigm. In the future it may be more rigorous, to invite all members of the expert panel to first attend a brief presentation about the model and its phases and then let them proceed to rate / classify. This way a shared understanding of each component of the model could be established.

These limitations do give way to the potential for future research. Future research should aim to extend the classification of the tools and techniques to the competencies or characteristics coaches require to effectively use such during the coaching process. Further, future research should not only be focused on the classification of tools/techniques to a given phase, but also on dissecting what specifically constitutes a positive psychological tool vs. a generic coaching strategy. Another aspect which needs to be considered is whether there is (or should be) a difference between the tools psychologists or therapists employ (e.g., psychological tools that are classified as psychological acts) and those that regular (non-psychologist) coaches interested in PPC could use in practice. A final matter that needs investigation is the empirical validation of the PPCM within a real-world environment.

## Conclusions

In conclusion, although Van Zyl et al. (10) addressed the need for an articulated definition and a process-orientated positive psychological coaching model, very little evidence existed in terms of clarity about techniques and tools explicitly applicable to PPC. Scientific knowledge should be available for other researchers to utilize, implement, validate, evaluate, critique, and update in an objective and systematic manner. With this article all four factors identified by George et al. (21) as important to operationalise a theoretical model have been theoretically confirmed, opening the way for further research and most importantly, empirical validation of the PPC model.

## Data Availability Statement

The raw data supporting the conclusions of this article will be made available by the authors, without undue reservation.

## Author Contributions

All authors contributed equally to conceptualization, data collection, analyses, and drafting of the manuscript.

## Conflict of Interest

The authors declare that the research was conducted in the absence of any commercial or financial relationships that could be construed as a potential conflict of interest.

## Footnotes

^1^Given the nature of the review, the effectiveness of the identified tools and techniques to promote positive psychological outcomes were not considered.
