# Supplementary material for: Positive Psychological Coaching Tools and Techniques: A Systematic Review and Classification
Source: Front Psychiatry. 2021 Jul 9;12:667200. doi: 10.3389/fpsyt.2021.667200 (PMC8298836; doi:10.3389/fpsyt.2021.667200)
Supplement: Supplementary file 1 [file Table_1.docx]

**Supplementary Material/Appendix.** **Articles included for qualitative synthesis.**

| **Authors** | **Year of Publication** | **Title of Paper** | **Publication** | **Type** | **Purpose** | **Citations** | **Reference** |
| --- | --- | --- | --- | --- | --- | --- | --- |
| A. Castiello D'Antonio | 2018 | Coaching psychology and positive psychology in work and organizational psychology | The Psychologist-Manager Journal | Scientific Journal | To examine the contribution of positive psychology to coaching psychology in the organizational contexts. | 9 | Castiello D'Antonio, A. (2018). Coaching psychology and positive psychology in work and organizational psychology. *The Psychologist-Manager Journal, 21*(2), 130-150. |
| A.M. Grant and G.B. Spence | 2010 | Using coaching and positive psychology to promote a flourishing workforce: A model of goal-striving and mental health | Oxford Handbook of positive psychology and work | Peer-Reviewed Chapter | To outline a range of coaching applications that are frequently used in the workplace and discusses differences | 47 | Grant, A.M., & Spence, G.B. (2010). Using coaching and positive psychology to promote a flourishing workforce: A model of goal-striving and mental health. In P.A. Linley, S. Harrington, & N. Page (Eds.), *Oxford Handbook of positive psychology and work* (pp. 175-188). Oxford: Oxford University Press. |
| C. Kauffman | 2006 | Positive Psychology: The Science at the Heart of Coaching | Evidence based coaching handbook: Putting best practices to work for your clients | Peer-Reviewed Chapter | To present a number of emerging trends in positive psychology theory and research and explore their applications to coaching | 216 | Kauffman, C. (2006). Positive Psychology: The Science at the Heart of Coaching. In D. R. Stober & A. M. Grant (Eds.), *Evidence based coaching handbook: Putting best practices to work for your clients* (pp. 219-253). Hoboken, NJ, US: John Wiley & Sons, Inc. |
| C. Kauffman and A. Scouler | 2004 | Toward a positive psychology of executive coaching | Positive psychology in practice | Peer-Reviewed Chapter | To describe the theoretical orientations of coaching, examine their relations with applications of positive psychology, and outline potential contents of a positive psychology model of coaching | 131 | Kauffman, C., & Scouler, A. (2004) Toward a positive psychology of executive coaching. In A. Linley & S. Josephs (Eds.), *Positive psychology in practice* (pp. 287–302)*.* Hoboken, NJ: John Wiley & Sons, Inc. |
| C. Kauffman, S. Joseph and A. Scoular | 2015 | Leadership coaching and positive psychology | Positive Psychology in Practice: Promoting Human Flourishing in Work, Health, Education, and Everyday Life | Scientific Journal | To describe the theoretical orientations of coaching, examine their relations with applications of positive psychology, and outline potential contents of a positive psychology model of coaching | 6 | Kauffman, C., Joseph, S., & Scoular, A. (2015). Leadership coaching and positive psychology. *Positive Psychology in Practice: Promoting Human Flourishing in Work, Health, Education, and Everyday Life*, 377-390. |
| C.M. Sims | 2017 | Second wave positive psychology coaching difficult emotions: Introducing the mnemonic of' ‘TEARS HOPE’ | The Coaching Psychologist | Scientific Journal | To illustrate the importance to maintain balance between the dark sides (weaknesses) along with the bright side (strengths) and to integrate the challenging and difficult aspects of human experience into our understanding of wellbeing and flourishing | 8 | Sims, C. M. (2017). Second wave positive psychology coaching difficult emotions: Introducing the mnemonic of' ‘TEARS HOPE’. *The Coaching Psychologist, 13*(2), 66-79. |
| D. N. Noble, K. Perkins and M. Fatout | 2000 | On being a strength coach: Child welfare and the strengths model | Child and Adolescent Social Work Journal | Scientific Journal | To give examples of the strength coach approach in the context of child welfare | 51 | Noble, D. N., Perkins, K., & Fatout, M. (2000). On being a strength coach: Child welfare and the strengths model. *Child and Adolescent Social Work Journal, 17*(2), 141-153. |
| F.W. Stander | 2016 | Strength Coaching as an Enabler of Positive Athlete Outcomes in a Multi-cultural Sport Environment | Coaching Psychology: Meta-theoretical perspectives and applications in multicultural contexts | Peer-Reviewed Chapter | To introduce a practical strength-based coaching model that can be applied in the context of sport | 1 | Stander, F. W. (2016). Strength Coaching as an Enabler of Positive Athlete Outcomes in a Multi-cultural Sport Environment. In L. E. van Zyl, M. W. Stander, & A. Odendaal (Eds.). *Coaching Psychology: Meta-theoretical perspectives and applications in multicultural contexts* (pp. 279-298): Cham: Springer. |
| J. M. Yeager and K. H. Britton | 2017 | Positive psychology coaching for sports leaders | Positive Psychology in Sport and Physical Activity | Peer-Reviewed Chapter | To demonstrate how Executive coaches with practices grounded in positive psychology can help sports leaders establish organisational cultures that contribute to higher performance through greater wellbeing | 1 | Yeager, J. M., & Britton, K. H. (2017). Positive psychology coaching for sports leaders. In A. Brady & B. Grenville-Cleave (Eds.). *Positive Psychology in Sport and Physical Activity* (pp. 243-255): London, UK: Routledge. |
| J. Passmore and L. G. Oades | 2015 | Positive psychology techniques: positive case conceptualisation | The Coaching Psychologist | Scientific Journal | To discuss the skill of positive case conceptualisation as a positive psychological technique | 9 | Passmore, J., & Oades, L. G. (2015). Positive psychology techniques: positive case conceptualisation. *The Coaching Psychologist, 11*(1), 43-45. |
| J. Passmore and L.G. Oades, L.G. | 2014 | Positive psychology coaching: a model for coaching practice | The Coaching Psychologist | Peer-Reviewed Chapter | To discuss positive psychology coaching (PPC) as an approach suitable for use with coaching clients | 19 | Passmore, J., & Oades, L. G. (2014). Positive psychology coaching: a model for coaching practice. *The Coaching Psychologist, 10*(2), 68-70. |
| L. E. Van Zyl and M.W. Stander | 2013 | A strengths-based approach towards coaching in a multicultural environment | Interdisciplinary handbook of the person-centered approach | Peer-Reviewed Chapter | To develop a strengths-based coaching model conducive to a multicultural environment | 20 | Van Zyl, L. E., & Stander, M. W. (2013). A strengths-based approach towards coaching in a multicultural environment. In J. H. D. Cornelius-White, R. Motschnig-Pitrik, & M. Lux (Eds.), *Interdisciplinary handbook of the person-centered approach* (pp. 245-257). New York, NY: Springer. |
| L. E. van Zyl, R. Motschnig-Pitrik and M. W. Stander | 2016 | Exploring positive psychology and person-centred psychology in multi-cultural coaching | Coaching psychology: Meta-theoretical perspectives and applications in multicultural contexts | Peer-Reviewed Chapter | To contrast eclectic and purist theorising through examining similarities and differences between positive psychology and the person-centred approach as dominant paradigm perspectives within multi-cultural coaching | 9 | Van Zyl, L. E., Motschnig-Pitrik, R., & Stander, M. W. (2016). Exploring positive psychology and person-centred psychology in multi-cultural coaching. In L. E. van Zyl, M. W. Stander, & A. Odendaal (Eds.). *Coaching psychology: Meta-theoretical perspectives and applications in multicultural contexts* (pp. 315-355). Cham: Springer. |
| L. G. Oades, T. P. Crowe and M. Nguyen | 2009 | Leadership coaching transforming mental health systems from the inside out: The Collaborative Recovery Model as person-centred strengths based coaching psychology | International Coaching Psychology Review | Scientific Journal | To present the Collaborative Recovery Model (CRM) as a person-centred strengths based coaching framework | 63 | Oades, L. G., Crowe, T. P., & Nguyen, M. (2009). Leadership coaching transforming mental health systems from the inside out: The Collaborative Recovery Model as person-centred strengths based coaching psychology. *International Coaching Psychology Review, 4*(1), 25-36. |
| M. K. White and P. Barnett | 2014 | A five-step model of appreciative coaching: a positive process for remediation | Remediation in Medical Education | Peer-Reviewed Chapter | To share experience of remediation coaches, describe the psychological foundations of appreciative inquiry and discuss the coaching context, roles, and process | 4 | White, M. K., & Barnett, P. (2014). A five step model of appreciative coaching: a positive process for remediation. In Kalet, A., & Chou, C. L. (Eds.) *Remediation in Medical Education* (pp. 265-281). New York, NY: Springer. |
| M. Tarragona | 2015 | Positive psychology and life coaching | Positive Psychology in Practice: Promoting Human Flourishing in Work, Health, Education, and Everyday Life | Peer-Reviewed Chapter | To define life coaching and what sets it apart from executive coaching and psychotherapy | 2 | Tarragona, M. (2015). Positive psychology and life coaching. In S. Joseph (Ed.), *Positive Psychology in Practice* (pp. 249-264). Hoboken, NJ: John Wiley & Sons. |
| M.B. Frisch | 2013 | Evidence-based wellbeing/positive psychology assessment and intervention with quality of life therapy and coaching and the Quality of Life Inventory (QOLI) | Social Indicators Research | Scientific Journal | To describe Quality of Life Therapy and Coaching and review developments and research since the publication of the manual in 2006 | 75 | Frisch, M. B. (2013). Evidence-based wellbeing/positive psychology assessment and intervention with quality of life therapy and coaching and the Quality of Life Inventory (QOLI). *Social Indicators Research, 114*(2), 193-227. |
| P. A. Linley, L. Woolston and R. Biswas-Diener | 2009 | Strengths coaching with leaders | International Coaching Psychology Review | Scientific Journal | To show how positive psychology, strengths approaches, and coaching psychology contribute to leadership strengths coaching programmes and practices | 272 | Linley, P. A., Woolston, L., & Biswas-Diener, R. (2009). Strengths coaching with leaders. *International Coaching Psychology Review, 4*(1), 37-48. |
| P.A. Linley and C. Kauffman | 2007 | Positive coaching psychology: Integrating the science of positive psychology with the practice of coaching psychology | International Coaching Psychology Review | Scientific Journal | To provide some perspectives from empirical and theoretical work at the intersection of positive psychology and coaching psychology | 4 | Linley, P. A., & Kauffman, C. (2007). Positive coaching psychology: Integrating the science of positive psychology with the practice of coaching psychology. *International Coaching Psychology Review, 2*(1), 5-8. |
| S. Gordon | 2016 | Strengths-based coaching: Case of mental toughness | Sports and athletics preparation, performance, and psychology. The psychology of effective coaching and management | Peer-Reviewed Chapter | To illustrate how a strengths-based approach can be used to coach behaviours | 3 | Gordon, S. (2016). Strengths-based coaching: Case of mental toughness. In P. A. Davis (Ed.), *Sports and athletics preparation, performance, and psychology. The psychology of effective coaching and management* (pp. 267–283). Hauppauge, New York: Nova Science Publishers. |
| S. Gordon and D.F. Gucciardi | 2011 | A strengths-based approach to coaching mental toughness | Journal of Sport Psychology in Action | Scientific Journal | To describe the history of the strengths-based approach and how to translate it into practice | 62 | Gordon, S., & Gucciardi, D. F. (2011). A strengths-based approach to coaching mental toughness. *Journal of Sport Psychology in Action, 2*(3), 143-155. |
| S. M. Dyess, R. Sherman, A. Opalinski and T. Eggenberger | 2017 | Structured coaching programs to develop staff | The Journal of Continuing Education in Nursing | Scientific Journal | To reflect on three coaching programs: Gallup Strengths-Based Coaching, Dartmouth Microsystem Coaching, and Health and Wellness Nurse Coaching | 11 | Dyess, S. M., Sherman, R., Opalinski, A., & Eggenberger, T. (2017). Structured coaching programs to develop staff. *The Journal of Continuing Education in Nursing, 48*(8), 373-378. |
| T. Anstiss and J. Passmore | 2017 | Wellbeing coaching | The Routledge companion to wellbeing at work | Peer-Reviewed Chapter | To combine insights from the science of wellbeing and positive psychology with coaching, to help coaches become as effective as possible in helping their clients take steps to protect and improve their wellbeing. | 4 | Anstiss, T., & Passmore, J. (2017). *Wellbeing coaching.* In C. Cooper & M. Leiter (Eds.), The Routledge companion to wellbeing at work (pp. 237-248). London, UK: Routledge. |
| T. Freire | 2013 | Positive psychology approaches | The Wiley-Blackwell Handbook of the Psychology of Coaching and Mentoring | Peer-Reviewed Chapter | To discuss how positive psychology science can be applied to coaching conceptualization and practices | 15 | Freire, T. (2013). Positive psychology approaches. In J. Passmore, D. Peterson, & T. Freire (Eds.), *The Wiley-Blackwell Handbook of the Psychology of Coaching and Mentoring* (pp. 426–442). West Sussex: Wiley-Blackwell. |
